# Neuroinflammatory response to lipopolysaccharide is exacerbated in mice genetically deficient in cyclooxygenase-2

**DOI:** 10.1186/1742-2094-5-17

**Published:** 2008-05-19

**Authors:** Saba Aid, Robert Langenbach, Francesca Bosetti

**Affiliations:** 1 Brain Physiology and Metabolism Section, National Institute on Aging, NIH, 9000 Memorial Drive, Bldg 9 Room 1S126, Bethesda, MD 20892, USA; 2 Laboratory of Molecular Carcinogenesis, National Institutes of Environmental Health, Research Triangle Park, NC 27709, USA

## Abstract

**Background:**

Cyclooxygenases (COX) -1 and -2 are key mediators of the inflammatory response in the central nervous system. Since COX-2 is inducible by inflammatory stimuli, it has been traditionally considered as the most appropriate target for anti-inflammatory drugs. However, the specific roles of COX-1 and COX-2 in modulating a neuroinflammatory response are unclear. Recently, we demonstrated that COX-1 deficient mice show decreased neuroinflammatory response and neuronal damage in response to lipopolysaccharide (LPS).

**Methods:**

In this study, we investigated the role of COX-2 in the neuroinflammatory response to intracerebroventricular-injected LPS (5 μg), a model of direct activation of innate immunity, using COX-2 deficient (COX-2^-/-^) and wild type (COX-2^+/+^) mice, as well as COX-2^+/+ ^mice pretreated for 6 weeks with celecoxib, a COX-2 selective inhibitor.

**Results:**

Twenty-four hours after LPS injection, COX-2^-/- ^mice showed increased neuronal damage, glial cell activation, mRNA and protein expression of markers of inflammation and oxidative stress, such as cytokines, chemokines, iNOS and NADPH oxidase. Brain protein levels of IL-1β, NADPH oxidase subunit p67^phox^, and phosphorylated-signal transducer and activator of transcription 3 (STAT3) were higher in COX-2^-/- ^and in celecoxib-treated mice, compared to COX-2^+/+ ^mice. The increased neuroinflammatory response in COX-2^-/- ^mice was likely mediated by the upregulation of STAT3 and suppressor of cytokine signaling 3 (SOCS3).

**Conclusion:**

These results show that inhibiting COX-2 activity can exacerbate the inflammatory response to LPS, possibly by increasing glial cells activation and upregulating the STAT3 and SOCS3 pathways in the brain.

## Introduction

Prostaglandin endoperoxide synthases or cyclooxygenases (COX-1 and COX-2) play a central role in the inflammatory cascade by converting arachidonic acid (AA), released from membrane phospholipids by a phospholipase A_2 _(PLA_2_), into prostaglandin endoperoxide H_2_, which in turn is converted to bioactive prostanoids by specific terminal synthases. The two COX isoforms share 60% homology in their amino acids sequence and have comparable kinetics; however they also show individual differences. COX-1 is normally constitutively expressed in most tissues and thought to be involved in homeostasis, whereas COX-2 is inducible upon inflammatory and other stimuli [1]. However, in the central nervous system (CNS), COX-1 and COX-2 are both constitutively expressed and COX-2 is mainly detected in the perinuclear, dendritic and axonal domains of neurons, particularly in cortex, hippocampus, amygdala and dorsal horn of the spinal cord of both rodent and human CNS [2-4]. In the CNS, COX-2 has been implicated in important physiological functions such as synaptic transmission, neurotransmitter release, blood flow regulation, and sleep/wake cycle [5-9].

Both COX-1 and COX-2 have been shown to play important roles in an inflammatory response, their contribution being different depending on the type of insult, the time after insult, and the tissue examined [6,10]. Because COX-2 is highly inducible by inflammatory stimuli it has been traditionally considered as the most appropriate target for anti-inflammatory drugs [2,11]. However, the exact role of each COX isoform in neuroinflammation is unclear. While we have recently reported that genetic deletion or pharmacological inhibition of COX-1 significantly ameliorate the neuroinflammatory response and brain injury following lipopolysaccharide (LPS) treatment [12], the role of COX-2 in the neuroinflammatory process remains controversial. For instance, COX-2 deficient (COX-2^-/-^) mice have been reported to be resistant to the febrile response induced by peripheral injection of LPS [13]. On the other hand, selective pharmacological inhibition of COX-2, but not of COX-1, increases the expression of several pro-inflammatory genes in the vascular associated cells and the parenchymal microglia after systemic injection of LPS [14].

In this study we examined the neuroinflammatory response of COX-2^-/- ^and wild type (COX-2^+/+^) mice to intracerebroventricular (icv) injection of LPS, which is a model of direct activation of brain innate immunity [15-19].

LPS, a component of the outer cell wall of gram-negative bacteria, mediates its effect through the CD14 receptor, a glycosylphosphatidylinositol-linked membrane protein that is present on microglial cells. The LPS-CD14 complex, together with other adaptor proteins, binds to the toll-like receptor 4 (TLR4), which is present on microglia, but not on astrocytes, oligodendrocytes or cortical neurons [20]. This initiates a bifurcated signal transduction cascade that leads to the transcription of inflammatory and immune response genes, primarily *via *nuclear factor- κB (NF-κB) activation but also through c-Fos/c-Jun and Janus kinase (JAK)/signal transducer and activator of transcription 3 (STAT3)-dependent pathways [21]. The signaling events ultimately lead to the production of free radicals generated by NADPH oxidase, myeloperoxidase and inducible nitric oxide synthase (iNOS) in combination with cytokines and chemokines [22,23], which are mediators of the LPS-induced injury [15,16,24]. In this regard, previous data suggest that interleukin-1β (IL-1β) and tumor necrosis factor-α (TNF-α) can contribute to neuronal death in models of acute CNS injury as well as in chronic neurodegenerative disease [25].

In this study, we demonstrate that COX-2^-/- ^mice are more susceptible than COX-2^+/+ ^mice to LPS-induced neuronal injury and exhibit an increase in microglia and astrocyte activation, and increases in the expression of genes and proteins for inflammatory cytokines, chemokines, reactive oxygen species-generating enzymes, such as iNOS and NADPH oxidase, and in the expression of STAT3 and suppressor of cytokine signaling 3 (SOCS3) signaling molecules. COX-2^+/+ ^mice chronically treated with celecoxib, a COX-2 selective inhibitor, also exhibit an increased neuroinflammatory response compared to untreated wild-type mice.

## Materials and methods

### Animals housing

Three-month-old male COX-2^+/+ ^and COX-2^-/- ^mice on a C57BL/6-129/Ola genetic background were used [26]. Mice were received at our animal facility at 6 weeks of age from a NIEHS colony maintained by Taconic Farms (Germantown, NY) with heterozygous by heterozygous breedings for greater than 35 generations. In order to prevent the inclusion of strain or genetic background confounders between COX deficient and wild type mice, all of the mice used in this study were progeny derived from heterozygous by heterozygous mating and therefore all contained the same strain and genetic background [26,27]. Mice were housed at 25°C in our animal facility with a 12 h light/dark cycle with free access to food and water. For celecoxib pretreatment, COX-2^+/+ ^mice were given free access for six weeks to a diet containing 0 or 6000 ppm celecoxib, a COX-2 specific inhibitor, as previously described [28]. Briefly, celecoxib (Celebrex™) capsules (400 mg; Pfizer Inc, New York, NY) were obtained from the NIH Division of Veterinary Medicine and were incorporated into feed by Research Diets, Inc. (New Brunswick, NJ). All procedures were performed under a NICHD approved animal protocol in accordance with NIH guidelines on the care and use of laboratory animals.

### LPS administration

Mice were anesthetized with ketamine (100 mg/kg) and xylazine (10 mg/kg, i.p.) and positioned in a stereotaxic apparatus (Kopf Instruments, Tujunga, CA). Vehicle (sterile phosphate buffer, 5 μl) or LPS (*E. coli *serotype 055:B5 (Sigma); 5 μg in 5 μl of sterile saline) was administered into the cerebral lateral ventricle using a 10 μl syringe with a 33 gauge needle (World Precision Instruments, Sarasota, FL) and a syringe pump (Stoelting, Wood Dale, IL) at a rate of 1 μl/min. This dose of LPS has been shown by us and by others to produce significant neuroinflammation when measured at 24 h [12,24,29]. The coordinates for the sterotaxic injections were -2.3 mm dorsal/ventral, -1.0 mm lateral, and -0.5 mm anterior/posterior from the bregma [30]. The needle was kept in this position for an additional 5 min after injection and then retrieved slowly from the brain.

### Tissue preparation and histology

Twenty-four hours after LPS injection, mice were anesthetized with sodium pentobarbital (100 mg/kg, i.p.) and then rapidly perfused transcardially with 0.9% saline solution containing 0.5% sodium nitrate and heparin (10 U/ml), followed by ice cold 4% paraformaldehyde in 0.1 M phosphate buffer. After transcardiac perfusions, brains were rapidly removed, postfixed for 4 h, and then cryoprotected in 30% sucrose at 4°C. Frozen brains were cut into 30 μm coronal sections using a cryostat and stored at -20°C.

Neurodegeneration was assessed using Fluoro-Jade B (FJB), as previously described [31]. Briefly, mounted brain sections were dried for 4 h, rehydrated through graded concentrations of alcohol (100, 70%; 1 min each), and rinsed for 1 min in distilled water. Sections were dipped and shaken in potassium permanganate (0.06%) for 20 min, rinsed for 1 min in distilled water, dipped, and shaken in a solution containing 0.004% FJB (Histochem, Jefferson, AR), 0.1% acetic acid for 20 min. The slides were thereafter rinsed three times in distilled water (1 min each), dried, dipped in xylene, and coverslipped with Permount mounting medium (Fisher Scientific, Ottawa, Ontario) [31].

For immunohistochemistry, free-floating sections were rinsed three times in phosphate-buffered saline (PBS) (10 min each) and then pretreated with PBS containing 3% hydrogen peroxide for 10 min to block endogenous peroxidase activity. After PBS wash, brain sections were incubated once in PBS with 0.3% Triton X-100 and once in PBS containing 0.5% BSA for 30 min with gentle shaking. The sections were incubated overnight at 4°C with anti-mouse scavenger receptor A (SRA) (1:100; Serotec, Raleigh, NC) in PBS containing 5% normal serum; followed by treatment with a biotinylated secondary antibody (Vector Laboratories, Burlingame, CA) for 1 h in PBS plus 5% normal serum at room temperature, and then with the Vector ABC kit (Vector Laboratories) for 1 h at room temperature. The sections were visualized with the 3,3-diaminobenzidine tetrachloride (DAB; Vector Laboratories, Burlingame, CA). Mounted brain sections were dried for 4 h, dehydrated through graded concentration of alcohol, cleared in xylene, and coverslipped with Permount mounting medium (Fisher Scientific).

### RNA extraction and quantitative real-time PCR

Brain total RNA was extracted using RNeasy Lipid Tissue Midi Kit (Qiagen, Valencia, CA, USA) as directed by the manufacturer. Total RNA extraction and reverse transcription were performed as previously described, using the Applied Biosystems Assay-On-Demand Gene Expression protocol with an ABI PRISM 7000 Sequence Detection System (Applied Biosystems, Foster City, CA) [32-34]. Briefly, five micrograms of total RNA were reverse transcribed using a High Capacity cDNA Archive kit (Applied Biosystems). Quantitative PCR for glial fibrillary acidic protein (GFAP), SRA1, TNF-α, IL-1β, interleukin 6 (IL-6), CCL2/monocyte chemoattractant protein-1 (MCP-1), CCL3/macrophage inflammatory protein 1 alpha (MIP-1α), iNOS, the NADPH oxidase subunits gp91^phox ^and p67^phox^, mPGES-1, cPLA_2_, COX-1, NF-κB-P65, STAT3, and SOCS3, was performed using specific Taqman^® ^probes (Applied Biosystems). Data were analyzed using the comparative threshold cycle (ΔΔ Ct) method [35]. Results were normalized with phosphoglycerate kinase 1 (Pgk1) as the endogenous control, and expressed as fold difference from the vehicle injected COX-2^+/+ ^mice.

### Western blotting

Western blot analyses were carried out as described previously [33] and nuclear proteins were prepared by using a compartmental protein extraction kit (Chemicon, Temecula) according to the manufacturer's protocol. Briefly, protein fractions were separated on Criterion gels (Bio-Rad), blotted onto a polyvinylidene difluoride membrane (Bio-Rad), and then immunoblotted with antibodies that recognize p67^phox ^(1:500; BD Biosciences), phosphorylated STAT3 (p-STAT3 (Tyr 705), 1:500, Cell signaling, USA), STAT3 (1:1000 Cell signaling, USA), COX-1 (1:500 Cayman Chemicals, USA), and glyceraldehyde dehydrogenase (GAPDH, 1: 2000, Santa Cruz, CA, USA) to control for protein loading. Blotted proteins were detected and quantified using an Odyssey Infrared Imaging System (Li-Cor Biosciences, Lincoln, NB, U.S.A.). For IL-1β measurement, a 500 μl aliquot of the crude brain homogenate was centrifuged at 10,000 × *g *for 20 min at 4°C, and the supernatant was immediately assayed using an ELISA-based kit (mouse IL-1β/IL-1F2 Quantikine ELISA kit, R&D Biosystems, Minneapolis, USA). TNF-α and MIP-1α were measured in the brain supernatant (Searchlight^® ^Sample testing service, Pierce Biotechnology, Woburn, MA, USA). Results were expressed as ng/g protein.

### Statistics

Data were expressed as mean ± SEM and were analyzed with a two-way ANOVA. For Real-Time PCR results, the two-way ANOVA was performed on the log-transformed ΔΔCt. p values < 0.05 were considered statistically significant.

## Results

### Only COX-2^-/- ^mice show FluorojadeB positive neurons 24 h after LPS

**Figure 1:**
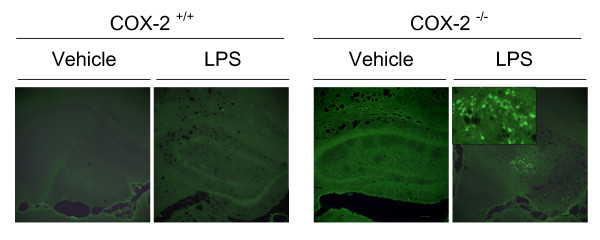
**Effects of COX-2 deficiency on LPS-induced neuronal degeneration.** Representative photomicrographs of FluoroJade-B staining in the hippocampus of COX-2^+/+ ^and COX-2^-/- ^mice 24 h after icv injection of LPS or vehicle. High magnification (10×) images of FluoroJade-B staining in the hippocampus are shown. The inset shows details of the neurons stained by FluoroJade-B in the hippocampus of COX-2^-/- ^mice 24 h after icv injection of LPS (40× magnification). Bars represent 100 μm.

To address the hypothesis that LPS exerts different neurotoxic effects in the COX-2^+/+ ^and COX-2^-/- ^mice, we assessed neuronal damage in the brain 24 h after LPS injection using the fluorescent marker FJB, which selectively stains injured neurons [31]. LPS-injected COX-2^-/- ^mice (Fig. 1) showed FJB-positive cells in the hippocampal area. In contrast, FJB-positive neurons were not detected in the hippocampus of vehicle-injected mice of each genotype and in LPS injected COX-2^+/+ ^mice.

### LPS-induced glial cell activation is increased in COX-2^-/- ^mice

**Figure 2:**
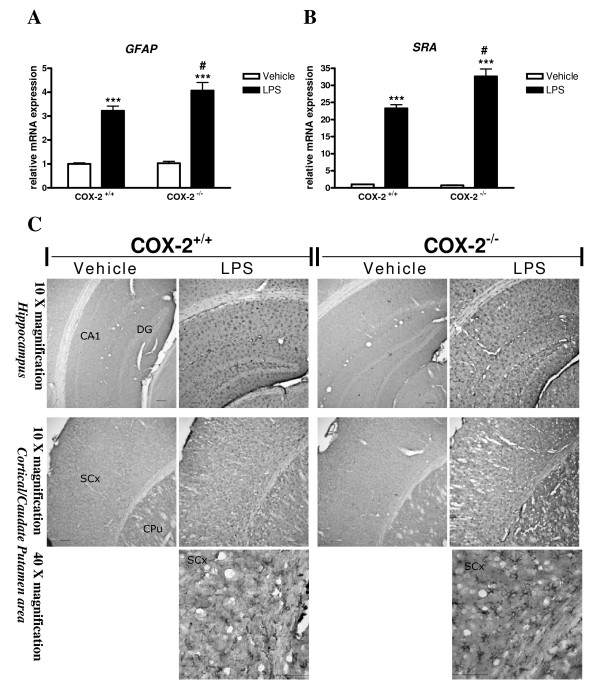
**Effects of COX-2 deficiency on LPS-induced expression of glial markers.** Quantitative real time-PCR analysis of astrocyte marker *GFAP *(A) and microglia marker *SRA *mRNA (B) in COX-2^+/+^and COX-2^-/- ^mice 24 h after icv injection of LPS or vehicle. Data are presented as mean ± SEM (*n *= 4-6). ***P < 0.001 compared to the corresponding vehicle-injected mice; ^#^*P *< 0.05 compared to the LPS-injected COX-2^+/+ ^mice. (C) Effects of COX-2 deficiency on LPS-induced activation of microglia. Representative photomicrographs of SRA immunohistochemistry in the striatum/caudate putamen and hippocampal area for COX-2^+/+ ^and COX-2^-/- ^mice 24 h after icv injection of LPS or vehicle. Bars represent 100 μm.

To determine glial cell response, we examined the expression of GFAP, a specific marker for astrocytes, and SRA, a specific marker for phagocytic microglia [36], 24 h after LPS injection, using quantitative real time PCR and immunohistochemistry. LPS markedly increased the expression of GFAP (Fig. 2A) and SRA mRNA (Fig. 2B) compared to vehicle injected mice, and the induction was higher in COX-2^-/- ^than in COX-2^+/+ ^mice.

Then we determined the immunoreactivity to SRA. In vehicle-treated COX-2^+/+ ^and COX-2^-/- ^mice, no SRA immunoreactivity was seen (Fig. 2C). Intense immunoreactive SRA-positive microglia with enhanced staining intensity, enlarged cell bodies, and thickening of processes were observed 24 h after LPS injection in the cortical/caudate putamen and hippocampal area of COX-2^+/+^mice. In LPS-injected COX-2^-/- ^mice, SRA-positive cells were numerous with higher cells retaining an enlarged cell body with thickening of ramified processes (Fig. 2C).

### Expression of cytokines and chemokines is increased in COX-2^-/- ^mice after LPS

**Figure 3:**
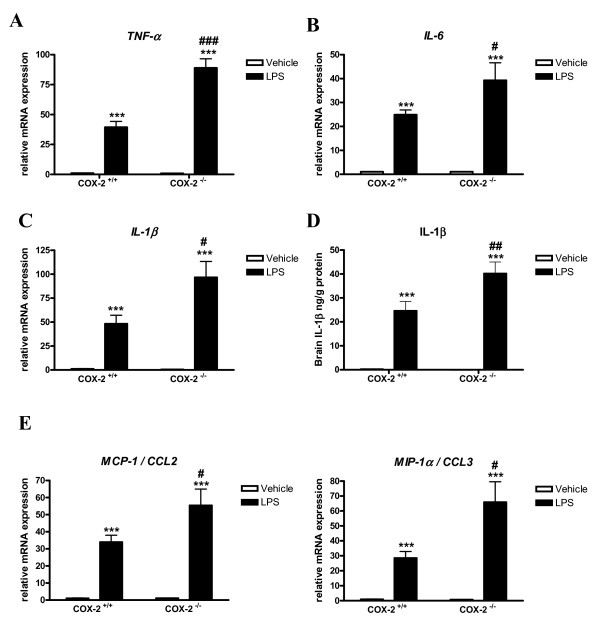
**Effects of COX-2 deficiency on LPS-induced expression of cytokines and chemokines.** Quantitative real time-PCR analysis of *TNF-α *(A), *IL-6 *(B), *IL-1β *(C), and CCL3/MIP-1α and CCL2/*MCP-1 *(E) for COX-2^+/+ ^and COX-2^-/- ^mice 24 h after icv injection of LPS or vehicle. (D) ELISA-based quantification of IL-1β protein levels in the brain of COX-2^+/+ ^and COX-2^-/-^mice 24 h after icv injection of LPS or vehicle. Data are presented as mean ± SEM (*n *= 4-6). ***P < 0.001 compared to the corresponding vehicle-injected mice; ^#^*P *< 0.05, ^##^*P *< 0.01, ^###^*P *< 0.001 compared to the LPS-injected COX-2^+/+ ^mice.

The expression of cytokine and chemokine genes, such as TNF-α, IL-6, IL-1β, MIP-1α/CCL3, MCP-1/CCL2, was determined quantitatively using real time PCR. LPS significantly increased the expression of TNF-α (Fig. 3A), IL-6 (Fig. 3B), IL-1β (Fig. 3C), MIP-1α and MCP-1 (Fig. 3E) mRNA in both COX-2^+/+ ^and COX-2^-/- ^mice. However, the mRNA levels of these cytokines were higher in the COX-2^-/-^compared to COX-2^+/+ ^mice (Fig. 3A–C and 3E). IL-1β was almost not detectable in the brain of vehicle-injected mice (Fig. 3D). Brain protein levels of IL-1β were also increased after LPS in both COX-2^+/+ ^and COX-2^-/- ^mice, and the increase was higher in the COX-2^-/- ^mice (Fig. 3D). Brain protein levels of TNF-α and of MIP-1α were also significantly increased after LPS injection in COX-2^-/-^compared to COX-2^+/+ ^mice (TNF-α: 3.9 ± 0.2, LPS injected COX-2^-/- ^(n = 5) vs. 2.2 ± 0.6 ng/g protein, LPS injected COX-2^+/+ ^(n = 3); MIP-1α: 340 ± 70, LPS injected COX-2^-/-^(n = 5) vs. 70 ± 41 ng/g protein, LPS injected COX-2^+/+ ^(n = 3), p < 0.05).

### mRNA expression of mPGES-1 is increased in COX-2^-/- ^mice after LPS

**Figure 4:**
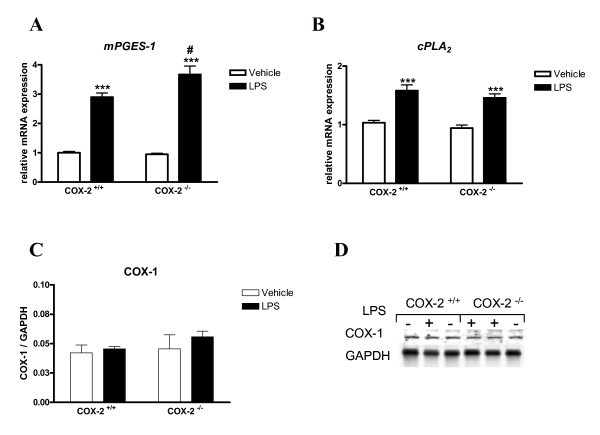
**Effects of COX-2 deficiency on LPS-induced expression of enzymes involved in the arachidonic acid cascade.** Quantitative real time-PCR analysis of *mPGES-1 *(A) and *cPLA*_2 _(B) for COX-2^+/+ ^and COX-2^-/- ^mice 24 h after icv injection of LPS or vehicle. (C) Quantification of COX-1 protein levels, relative to GAPDH internal loading control in the whole brain. (D) Representative immunoblot of COX-1 expression in COX-2^+/+ ^and COX-2^-/- ^mice 24 h after icv injection of LPS or vehicle. Data are presented as mean ± SEM (*n *= 4-6). ***P < 0.001 compared to the corresponding vehicle-injected mice; ^#^*P *< 0.05 compared to the LPS-injected COX-2^+/+ ^mice.

Brain mRNA levels of mPGES-1 were significantly increased by LPS, but the induction was significantly higher in the COX-2^-/- ^mice compared to COX-2^+/+ ^mice (Fig. 4A). In contrast, cPLA_2 _mRNA expression was increased by LPS to a similar extent in both COX-2^+/+ ^and in COX-2^-/- ^mice (Fig. 4B). COX-1 protein (Fig. 4C and 4D) and mRNA levels were not significantly changed by either LPS treatment or mouse genotype (COX-2^+/+ ^mice: 0.8 ± 0.03 LPS vs. 1.0 ± 0.07 vehicle; COX-2^-/- ^mice: 1.5 ± 0.41 LPS vs. 0.9 ± 0.07 vehicle; p > 0.05), suggesting that the enhanced neuroinflammatory response in COX-2^-/- ^mice is not mediated by a compensatory induction in COX-1 after LPS. The expression of cPGES and mPGES-2 mRNA was not significantly different between COX-2^+/+ ^and COX-2^-/- ^mice after LPS injection (data not shown), suggesting that these enzymes are not involved in the enhanced neuroinflammatory response of COX-2^-/- ^mice at the time point examined.

### Expression of reactive oxygen species generating enzymes is increased in COX-2^-/- ^mice after LPS

**Figure 5:**
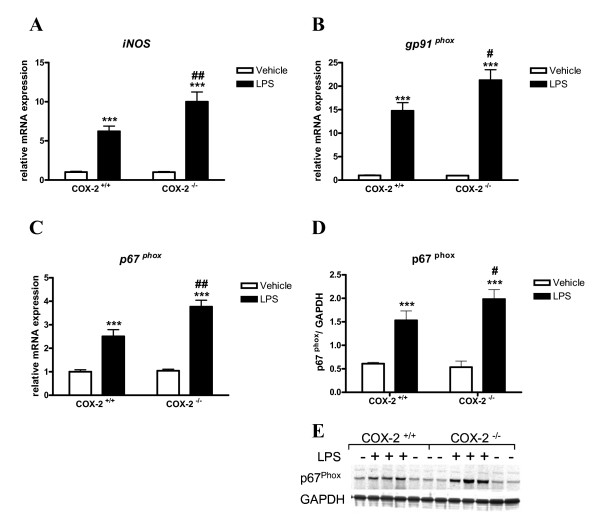
**Effects of COX-2 deficiency on LPS-induced expression of ROS-generating enzymes.** Quantitative real time-PCR analysis of *iNOS *(A), *gp91*^*phox *^*(*B) and *p67*^*phox *^mRNA (C) for COX-2^+/+ ^and COX-2^-/- ^mice that received icv injection of LPS or vehicle 24 h before sacrifice. (D) Quantification of p67^phox ^protein levels, relative to GAPDH internal loading control in the whole brain. (E) Representative immunoblot of p67^phox ^expression in COX-2^+/+ ^and COX-2^-/- ^mice 24 h after icv injection of LPS or vehicle. Data are presented as mean ± SEM (*n *= 4-6). ***P < 0.001 compared to the corresponding vehicle-injected mice; ^#^*P *< 0.05, ^##^*P *< 0.01 compared to the LPS-injected COX-2^+/+ ^mice.

iNOS and NADPH oxidase are major sources of reactive oxygen species during the inflammatory process, and both are expressed in glial cells. We examined gene and protein expression of ROS-generating enzymes in COX-2^-/- ^mice 24 h after LPS injection using quantitative real time-PCR and Western blotting. In response to LPS, COX-2^-/- ^mice showed a higher increase in iNOS mRNA levels compared to COX-2^+/+ ^mice (Fig. 5A). Similarly, mRNA levels of NADPH oxidase cytosolic subunit p67^phox ^and membrane subunit gp91^phox ^were significantly elevated at 24 h after LPS injection in both genotypes compared with vehicle-injected controls (Fig. 5B and 5C). COX-2^-/- ^mice showed a higher increase in the expression of p67^phox ^and gp91^phox ^mRNA than COX-2^+/+ ^mice (Fig. 5B and 5C). Western blotting analysis confirmed that protein level of the p67^phox ^subunit was higher in the COX-2^-/- ^mice compared to the COX-2^+/+ ^mice after LPS (Fig. 5D and 5E).

### Phosphorylated STAT3 and the mRNA expression of STAT3 and SOCS3, but not of NF-κB, are increased in COX-2^-/- ^mice after LPS

**Figure 6:**
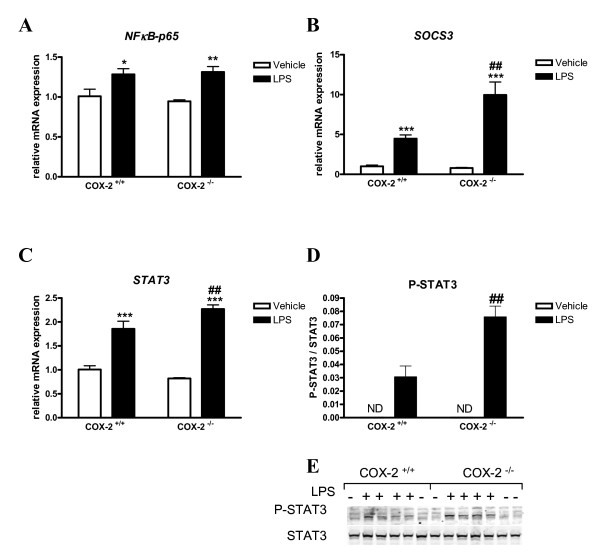
**Effects of COX-2 deficiency on LPS-induced expression of transcription factors NF-κB and STAT3, and SOCS3.** Quantitative real time-PCR analysis of *NF-κB *(A), *SOCS3 *(B) and *STAT3 *mRNA (C) for COX-2^+/+ ^and COX-2^-/- ^mice 24 h after icv injection of LPS or vehicle. (D) Quantification of phosphorylated STAT3 (Tyr 705) protein levels, relative to the total STAT3 in the brain nuclear fraction. (E) Representative immunoblot of P-STAT3 expression in COX-2^+/+ ^and COX-2^-/- ^mice 24 h after icv injection of LPS or vehicle. Data are presented as mean ± SEM (*n *= 4-6). *P < 0.05, **P < 0.01, ***P < 0.001 compared to the corresponding vehicle-injected mice; ^##^*P *< 0.01 compared to the LPS-injected COX-2^+/+ ^mice.

Activation of the transcription factors NF-κB and STAT3 plays a critical role in the production of inflammatory mediators by activated microglia [12]. STAT signaling is tightly regulated by its negative regulators, suppressors of cytokine signaling (SOCS), and is controlled by STAT phosphorylation [37]. To determine the transcriptional mechanism underlying the increased inflammatory response to LPS observed in the COX-2^-/- ^mice, we examined NF-κB p65 and STAT3 levels. The mRNA expression of NF-κB (Fig. 6A) was increased similarly in COX-2^+/+ ^and COX-2^-/- ^mice after LPS. The expression of STAT3 and SOCS3 mRNA was also significantly increased 24 h after LPS injection in both genotypes compared to the vehicle-injected COX-2^+/+ ^mice (Fig. 6B and 6C), but the increase was significantly higher in COX-2^-/- ^mice than in COX-2^+/+ ^mice. Phosphorylated STAT3 was not detected in the brain nuclear fraction of vehicle-injected mice (Fig. 6D and 6E); however, after LPS, phosphorylated STAT3 protein level increased in both genotypes, but was increased to a greater extent in COX-2^-/- ^mice (Fig. 6D and 6E).

### Pretreatment of COX-2^+/+ ^mice with celecoxib for 6 weeks increases LPS-induced brain IL-1β level, NADPH oxidase subunit p67^phox^, and phosphorylated STAT3

**Figure 7:**
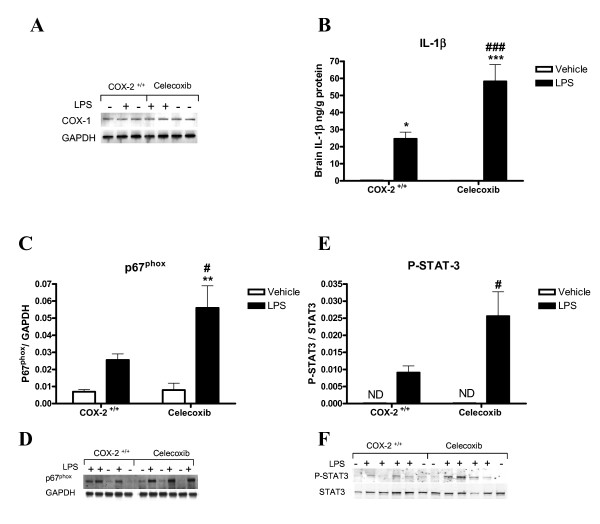
**Effects of chronic pretreatment with celecoxib (6000 ppm for 6 weeks) on LPS-induced brain IL-1β levels, the expression of COX-1, p67^phox ^NADPH oxidase subunit and the phosphorylated STAT3.** (A) Representative immunoblot of COX-1 protein levels, relative to GAPDH internal loading control, in the whole brain of COX-2^+/+ ^and celecoxib-treated COX-2^+/+ ^mice 24 h after icv injection of LPS or vehicle. (B) ELISA based immunoassay for brain IL-1β in celecoxib-treated mice (n = 7) and untreated COX-2^+/+ ^mice (n = 4). (C) Quantification of p67^phox ^protein levels, relative to GAPDH internal loading control in the whole brain with representative immunoblot (D) in celecoxib-treated mice and untreated COX-2^+/+ ^mice (n = 4). (E) Quantification of phosphorylated STAT3 (Tyr 705) protein levels, relative to the total STAT3 in the brain nuclear fraction and the representative immunoblot (F) in celecoxib-treated mice and untreated COX-2^+/+ ^mice (n = 4). All data are expressed as mean ± SEM. *P <0.05, **P < 0.01, ***P < 0.001 compared to the corresponding vehicle-injected mice; ^#^*P *< 0.05, ^###^*P *< 0.001 compared to the LPS-injected COX-2^+/+ ^mice.

Brain COX-1 protein levels were not significantly changed by LPS treatment either in COX-2^+/+ ^or in celecoxib-treated mice (Fig. 7A, COX-2^+/+ ^mice: 0.041 ± 0.009 LPS vs. 0.033 ± 0.003 vehicle; celecoxib-treated COX-2^+/+ ^mice: 0.043 ± 0.0.008 LPS vs. 0.038 ± 0.004 vehicle; p > 0.05). COX-2^+/+ ^mice pretreated with celecoxib showed a significant increase in brain IL-1β protein level after LPS, compared to untreated mice (Fig. 7B). The protein levels of p67^phox ^(Fig. 7C and 7D) and phosphorylated STAT3 after LPS (Fig. 7E and 7F) were also higher in the celecoxib-treated mice compared to untreated mice.

## Discussion

In this study we demonstrate for the first time that genetic deletion of COX-2 enhanced the neuroinflammatory response and increased the susceptibility to neuronal damage induced by centrally injected LPS. We also showed that chronic treatment with a selective COX-2 inhibitor, celecoxib, also increases LPS-induced protein levels of IL-1β, a major proinflammatory cytokine, of phosphorylated STAT3, a transcription factor involved in the progression of the inflammatory cascade, and of NADPH oxidase subunit p67^phox^, a marker of oxidative stress. We have previously demonstrated that this chronic dosing paradigm of celecoxib (6000 ppm for 6 weeks) leads to a plasma concentration of 18.2 ± 5.8 μg/ml [28]. Assuming 98% binding of celecoxib to plasma proteins and that only free celecoxib can cross the blood brain barrier [38], brain concentration of celecoxib is approximately 640 nM, well above the IC_50 _(39 nM) of celecoxib for COX-2 [28]. These plasma concentrations are within the same order of magnitude of steady state concentrations (2–3 μg/ml) observed in humans after acute administration of 400–800 mg of celecoxib, doses clinically used for the treatment of rheumatoid arthritis and familial adenomatous polyposis [39].

In this study, FJB-positive neurons were only observed in the COX-2^-/- ^mice, suggesting that COX-2 deletion increases the susceptibility to LPS-induced neurodegeneration. There is a conflicting view about the role of COX-2 in neurodegeneration and neurotoxicity [40]. For instance, COX-2 inhibition is believed to be neuroprotective in models such as MPTP (1-methyl 4-phenyl 1,2,3,6-tetrahydropyridine), quisquallic acid induced damage [41,42], and centrally injected NMDA-induced neurotoxicity [43,44]. However, in these studies, where the toxins directly damage neurons, COX-2-mediated cytotoxicity does not appear to be linked to the inflammatory response [41]. On the other hand, pre-treatment with COX-2 inhibitors or genetic deletion of COX-2 has been shown to increase seizure activity and neuronal damage in response to kainate [28,45], and to exacerbate endotoxin-induced ocular inflammation [46] and tissue damage in ConA- and acetaminophen-induced hepatotoxicity [47,48]. Another study reported, in support of our observations, that selective pharmacological inhibition of COX-2 with NS-398 increases the transcription of inflammatory genes (mPGES-1, TLR2, CD14, MCP-1) in vascular associated brain cells and parenchymal microglia after systemic injection of LPS [14]. While these conflicting data highlight the importance of investigating the distinct roles of COX-1 and COX-2 in physiology and pathology, our findings suggest that COX-2-derived products selectively mediate a protective effect in the development and/or the resolution of inflammation in the brain after endotoxin activation of the innate immune system. In this regard, a recent review emphasizes that COX-2 mediates neuroprotection *via *specific anti-inflammatory lipid mediators [49]. Furthermore, Gilroy and colleagues demonstrated that selective COX-2 inhibitors, by blocking the production of PGE_2 _and PGD_2_, disturbed the resolution phase of inflammation, leading to delay in return to homeostasis [50].

COX-1 protein levels were not significantly changed by LPS in either COX-2^-/- ^or celecoxib-treated mice compared to COX-2^+/+ ^mice, indicating that the increased neuroinflammatory response was not due to an increased compensatory expression of COX-1 in response to LPS when COX-2 is either genetically abrogated or pharmacologically inhibited. Increases in microglial activation and in the induction of cytokines and chemokines in the COX-2^-/- ^mice could contribute to the susceptibility to LPS-induced damage. Overexpression of chemokines, small pleiotropic chemoattractant cytokines that promote leukocytes activation and migration, has been recently implicated in many neurological disorders including multiple sclerosis, and Alzheimer's disease [51,52]. The overexpression of chemokines observed in the COX-2^-/- ^mice after LPS may increase the leukocytes and monocytes recruitment in the inflamed brain and cause neuronal damages, in the absence of a "switch off" mechanism. The increased expression of cytokines could be due to the incapacity of the tissue to resolve the inflammation, leading to a persistent activation of the inflammatory cascade. One possibility is that COX-2 deletion or inhibition leads to a reduction in anti-inflammatory mediators or neurotrophic factors, which would impair the brain ability to resolve the inflammation.

iNOS and NADPH oxidase may also contribute to microglia-mediated LPS induced neurotoxicity by increasing the production of extracellular reactive oxygen and nitrogen species, which, in turn, stimulate the microglial release of pro-inflammatory mediators that, like radical oxygen species, are toxic to neurons [53,54]. In this regard, NADPH inhibitors suppress LPS-induced expression of iNOS, IL-6, IL-1β and TNF-α in glial cells *in vitro *[55] and NADPH oxidase has been shown to regulate COX-2 mediated PGE_2 _production in cultured microglia [56].

The JAK/STAT pathway is a key player in the intracellular response to cytokines. SOCS3 is a potent inhibitor of the JAK/STAT signaling cascade, negatively regulating signal transduction pathways mediated by a variety of cytokines. SOCS3 has been suggested to play a critical role in integrating the neuroimmunoendocrine circuits [57]. Although NF-κB p65 expression were similar in COX-2^+/+ ^and COX-2^-/- ^mice after LPS, we found that the mRNA expression of STAT3 and the levels of phosphorylated STAT-3 were significantly higher in the COX-2^-/- ^mice compared to wild type mice. SOCS3 was also upregulated in the COX-2^-/- ^mice compared to COX-2^+/+ ^mice after LPS. SOCS3 is a negative modulator of inflammatory cytokine signaling [58] and can be induced by inflammatory stimuli such as LPS, TNF-α and IL-6 [58,59]. SOCS3 mRNA up-regulation in the COX-2 deficient mice can thus be viewed as the consequence of the higher cytokine production in these mice after LPS. In this regard, SOCS3 overexpression has been shown to lead to neuroblastoma cell death [60]. Overall, our data indicate a dysregulation of the cytokine signaling pathway in the COX-2^-/- ^mice, which may mediate the increased neuroinflammatory response.

While independent epidemiological studies indicate that non steroidal anti-inflammatory drugs (NSAIDs) administration prevents or delays the onset and risk of developing Alzheimer's disease [61-63], clinical trials using COX-2 selective inhibitors in patients with mild to severe cognitive impairment, have been unsuccessful to date [64-67], with the exception of a small double blind, placebo-controlled study with indomethacin, a preferential COX-1 inhibitor [68]. We have recently demonstrated that genetic deletion or pharmacological inhibition of COX-1 significantly attenuates glial cells activation and the neuroinflammatory response, oxidative stress and neuronal damage in response to icv injected LPS [12]. Our results show that while COX-1 selective inhibition may be beneficial, selective inhibition of COX-2 appears not to be beneficial in neurodegenerative diseases with a marked inflammatory component and may explain the failure of selective COX-2 inhibitors to protect AD patients from cognitive decline in clinical trials [64-67].

## Conclusion

These findings altogether indicate that the two COX isoforms display opposite roles in the brain during the acute neuroinflammatory process and that COX-2 inhibition worsens the inflammatory response to LPS, suggesting a neuroprotective function of COX-2-derived products. In this regard, further investigations are warranted to identify which specific COX-2 products may mediate the neuroprotective effects and more research should be focused on COX-1 selective inhibitors for the treatment of neurological and neurodegenerative diseases with an inflammatory component.

## Abbreviations

AD: Alzheimer's disease; ANOVA: analysis of variance; BSA: bovine serum albumin; COX: cyclooxygenase; CNS: central nervous system; DAB: 3,3-diaminobenzidine tetrachloride; ELISA: enzyme linked immunosorbent assay; GAPDH: glyceraldehyde dehydrogenase; GFAP: glial fibrillary acidic protein; icv: intracerebroventricular; IL-1β: interleukin1 beta; IL-6: interleukin 6; iNOS: inducible nitric oxide synthase; JAK: Janus kinase; LPS: lipopolysaccharide; MCP-1: monocyte chemoattractant protein-1; MIP-1α: macrophage inflammatory protein 1 alpha; mPGES-1 or -2: microsomal prostaglandin E synthase-1 or -2; cPGES: cytosolic prostaglandin E synthase; NF-κB: nuclear factor- κB; PBS: phosphate-buffered saline; PGD_2_: prostaglandin D_2_; PGE_2_: prostaglandin E_2_; PLA_2_: phospholipase A_2_; ROS: reactive oxygen species; SOCS3: suppressors of cytokine signaling 3; SRA: scavenger receptor A; STAT3: signal transducer and activator of transcription 3; TNF-α: Tumor Necrosis Factor alpha.

## Competing interests

The authors declare that they have no competing interests.

## Authors' contributions

SA performed the experiments, contributed to study design and data analysis and wrote the manuscript. RL provided the COX-2^+/+ ^and COX-2^-/-^mice and reviewed the manuscript. FB directed the work, contributed to study design, reviewed the data, and wrote the manuscript. All authors read and approved the final manuscript.

## References

[B1] Morita I (2002). Distinct functions of COX-1 and COX-2. Prostaglandins Other Lipid Mediat.

[B2] Kaufmann WE, Andreasson KI, Isakson PC, Worley PF (1997). Cyclooxygenases and the central nervous system. Prostaglandins.

[B3] Yamagata K, Andreasson KI, Kaufmann WE, Barnes CA, Worley PF (1993). Expression of a mitogen-inducible cyclooxygenase in brain neurons: regulation by synaptic activity and glucocorticoids. Neuron.

[B4] Breder CD, Dewitt D, Kraig RP (1995). Characterization of inducible cyclooxygenase in rat brain. J Comp Neurol.

[B5] Hayaishi O, Matsumura H (1995). Prostaglandins and sleep. Adv Neuroimmunol.

[B6] Minghetti L (2007). Role of COX-2 in inflammatory and degenerative brain diseases. Subcell Biochem.

[B7] Kaufmann WE, Worley PF, Pegg J, Bremer M, Isakson P (1996). COX-2, a synaptically induced enzyme, is expressed by excitatory neurons at postsynaptic sites in rat cerebral cortex. Proc Natl Acad Sci USA.

[B8] Stefanovic B, Bosetti F, Silva AC (2006). Modulatory role of cyclooxygenase-2 in cerebrovascular coupling. Neuroimage.

[B9] Ojeda SR, Urbanski HF, Junier MP, Capdevila J (1989). The role of arachidonic acid and its metabolites in the release of neuropeptides. Ann N Y Acad Sci.

[B10] Graham SH, Hickey RW (2003). Cyclooxygenases in central nervous system diseases: a special role for cyclooxygenase 2 in neuronal cell death. Arch Neurol.

[B11] Phillis JW, Horrocks LA, Farooqui AA (2006). Cyclooxygenases, lipoxygenases, and epoxygenases in CNS: their role and involvement in neurological disorders. Brain Res Rev.

[B12] Choi SH, Langenbach R, Bosetti F (2008). Genetic deletion or pharmacological inhibition of cyclooxygenase-1 attenuate lipopolysaccharide-induced inflammatory response and brain injury. FASEB J.

[B13] Li S, Wang Y, Matsumura K, Ballou LR, Morham SG, Blatteis CM (1999). The febrile response to lipopolysaccharide is blocked in cyclooxygenase-2(-/-), but not in cyclooxygenase-1(-/-) mice. Brain Res.

[B14] Blais V, Turrin NP, Rivest S (2005). Cyclooxygenase 2 (COX-2) inhibition increases the inflammatory response in the brain during systemic immune stimuli. J Neurochem.

[B15] Milatovic D, VanRollins M, Li K, Montine KS, Montine TJ (2005). Suppression of murine cerebral F2-isoprostanes and F4-neuroprostanes from excitotoxicity and innate immune response in vivo by alpha- or gamma-tocopherol. J Chromatogr B Analyt Technol Biomed Life Sci.

[B16] Milatovic D, Zaja-Milatovic S, Montine KS, Shie FS, Montine TJ (2004). Neuronal oxidative damage and dendritic degeneration following activation of CD14-dependent innate immune response in vivo. J Neuroinflammation.

[B17] Bluthe RM, Laye S, Michaud B, Combe C, Dantzer R, Parnet P (2000). Role of interleukin-1beta and tumour necrosis factor-alpha in lipopolysaccharide-induced sickness behaviour: a study with interleukin-1 type I receptor-deficient mice. Eur J Neurosci.

[B18] Abdulla D, Goralski KB, Del Busto Cano EG, Renton KW (2005). The signal transduction pathways involved in hepatic cytochrome P450 regulation in the rat during a lipopolysaccharide-induced model of central nervous system inflammation. Drug Metab Dispos.

[B19] Montine TJ, Milatovic D, Gupta RC, Valyi-Nagy T, Morrow JD, Breyer RM (2002). Neuronal oxidative damage from activated innate immunity is EP2 receptor-dependent. J Neurochem.

[B20] Lehnardt S, Massillon L, Follett P, Jensen FE, Ratan R, Rosenberg PA, Volpe JJ, Vartanian T (2003). Activation of innate immunity in the CNS triggers neurodegeneration through a Toll-like receptor 4-dependent pathway. Proc Natl Acad Sci USA.

[B21] Hosoi T, Okuma Y, Kawagishi T, Qi X, Matsuda T, Nomura Y (2004). Bacterial endotoxin induces STAT3 activation in the mouse brain. Brain Res.

[B22] Quan N, Sundar SK, Weiss JM (1994). Induction of interleukin-1 in various brain regions after peripheral and central injections of lipopolysaccharide. J Neuroimmunol.

[B23] Luster AD (1998). Chemokines – chemotactic cytokines that mediate inflammation. N Engl J Med.

[B24] Milatovic D, Zaja-Milatovic S, Montine KS, Horner PJ, Montine TJ (2003). Pharmacologic suppression of neuronal oxidative damage and dendritic degeneration following direct activation of glial innate immunity in mouse cerebrum. J Neurochem.

[B25] Perry VH, Newman TA, Cunningham C (2003). The impact of systemic infection on the progression of neurodegenerative disease. Nat Rev Neurosci.

[B26] Morham SG, Langenbach R, Loftin CD, Tiano HF, Vouloumanos N, Jennette JC, Mahler JF, Kluckman KD, Ledford A, Lee CA, Smithies O (1995). Prostaglandin synthase 2 gene disruption causes severe renal pathology in the mouse. Cell.

[B27] Toscano CD, Prabhu VV, Langenbach R, Becker KG, Bosetti F (2007). Differential gene expression patterns in cyclooxygenase-1 and cyclooxygenase-2 deficient mouse brain. Genome Biol.

[B28] Toscano CD, Ueda Y, Tomita YA, Vicini S, Bosetti F (2008). Altered GABAergic neurotransmission is associated with increased kainate-induced seizure in prostaglandin-endoperoxide synthase-2 deficient mice. Brain Res Bull.

[B29] Goralski KB, Abdulla D, Sinal CJ, Arsenault A, Renton KW (2005). Toll-like receptor-4 regulation of hepatic Cyp3a11 metabolism in a mouse model of LPS-induced CNS inflammation. Am J Physiol Gastrointest Liver Physiol.

[B30] Paxinos G, Franklin KBJ, (Eds.) (2001). The mouse brain in stereotaxic coordinates.

[B31] Schmued LC, Hopkins KJ (2000). Fluoro-Jade B: a high affinity fluorescent marker for the localization of neuronal degeneration. Brain Res.

[B32] Bosetti F, Langenbach R, Weerasinghe GR (2004). Prostaglandin E2 and microsomal prostaglandin E synthase-2 expression are decreased in the cyclooxygenase-2-deficient mouse brain despite compensatory induction of cyclooxygenase-1 and Ca2+-dependent phospholipase A2. J Neurochem.

[B33] Choi SH, Langenbach R, Bosetti F (2006). Cyclooxygenase-1 and -2 enzymes differentially regulate the brain upstream NF-kappa B pathway and downstream enzymes involved in prostaglandin biosynthesis. J Neurochem.

[B34] Aid S, Bosetti F (2007). Gene expression of cyclooxygenase-1 and Ca(2+)-independent phospholipase A(2) is altered in rat hippocampus during normal aging. Brain Res Bull.

[B35] Livak KJ, Schmittgen TD (2001). Analysis of relative gene expression data using real-time quantitative PCR and the 2(-Delta Delta C(T)) Method. Methods.

[B36] Herber DL, Maloney JL, Roth LM, Freeman MJ, Morgan D, Gordon MN (2006). Diverse microglial responses after intrahippocampal administration of lipopolysaccharide. Glia.

[B37] Planas AM, Gorina R, Chamorro A (2006). Signalling pathways mediating inflammatory responses in brain ischaemia. Biochem Soc Trans.

[B38] Dembo G, Park SB, Kharasch ED (2005). Central nervous system concentrations of cyclooxygenase-2 inhibitors in humans. Anesthesiology.

[B39] Davies NM, McLachlan AJ, Day RO, Williams KM (2000). Clinical pharmacokinetics and pharmacodynamics of celecoxib: a selective cyclo-oxygenase-2 inhibitor. Clin Pharmacokinet.

[B40] Bosetti F (2007). Arachidonic acid metabolism in brain physiology and pathology: lessons from genetically altered mouse models. J Neurochem.

[B41] Teismann P, Tieu K, Choi DK, Wu DC, Naini A, Hunot S, Vila M, Jackson-Lewis V, Przedborski S (2003). Cyclooxygenase-2 is instrumental in Parkinson's disease neurodegeneration. Proc Natl Acad Sci USA.

[B42] Scali C, Prosperi C, Vannucchi MG, Pepeu G, Casamenti F (2000). Brain inflammatory reaction in an animal model of neuronal degeneration and its modulation by an anti-inflammatory drug: implication in Alzheimer's disease. Eur J Neurosci.

[B43] Iadecola C, Niwa K, Nogawa S, Zhao X, Nagayama M, Araki E, Morham S, Ross ME (2001). Reduced susceptibility to ischemic brain injury and N-methyl-D-aspartate-mediated neurotoxicity in cyclooxygenase-2-deficient mice. Proc Natl Acad Sci USA.

[B44] Hewett SJ, Silakova JM, Hewett JA (2006). Oral treatment with rofecoxib reduces hippocampal excitotoxic neurodegeneration. J Pharmacol Exp Ther.

[B45] Baik EJ, Kim EJ, Lee SH, Moon C (1999). Cyclooxygenase-2 selective inhibitors aggravate kainic acid induced seizure and neuronal cell death in the hippocampus. Brain Res.

[B46] Tuo J, Tuaillon N, Shen D, Chan CC (2004). Endotoxin-induced uveitis in cyclooxygenase-2-deficient mice. Invest Ophthalmol Vis Sci.

[B47] Yin H, Cheng L, Langenbach R, Ju C (2007). Prostaglandin I(2) and E(2) mediate the protective effects of cyclooxygenase-2 in a mouse model of immune-mediated liver injury. Hepatology.

[B48] Reilly TP, Bourdi M, Brady JN, Pise-Masison CA, Radonovich MF, George JW, Pohl LR (2001). Expression profiling of acetaminophen liver toxicity in mice using microarray technology. Biochem Biophys Res Commun.

[B49] Serhan CN (2007). Resolution phase of inflammation: novel endogenous anti-inflammatory and proresolving lipid mediators and pathways. Annu Rev Immunol.

[B50] Gilroy DW, Colville-Nash PR, Willis D, Chivers J, Paul-Clark MJ, Willoughby DA (1999). Inducible cyclooxygenase may have anti-inflammatory properties. Nat Med.

[B51] Bajetto A, Bonavia R, Barbero S, Florio T, Schettini G (2001). Chemokines and their receptors in the central nervous system. Front Neuroendocrinol.

[B52] Savarin-Vuaillat C, Ransohoff RM (2007). Chemokines and chemokine receptors in neurological disease: raise, retain, or reduce?. Neurotherapeutics.

[B53] Block ML, Hong JS (2005). Microglia and inflammation-mediated neurodegeneration: multiple triggers with a common mechanism. Prog Neurobiol.

[B54] Babior BM (2004). NADPH oxidase. Curr Opin Immunol.

[B55] Pawate S, Shen Q, Fan F, Bhat NR (2004). Redox regulation of glial inflammatory response to lipopolysaccharide and interferongamma. J Neurosci Res.

[B56] Wang T, Qin L, Liu B, Liu Y, Wilson B, Eling TE, Langenbach R, Taniura S, Hong JS (2004). Role of reactive oxygen species in LPS-induced production of prostaglandin E2 in microglia. J Neurochem.

[B57] Auernhammer CJ, Melmed S (2001). The central role of SOCS-3 in integrating the neuro-immunoendocrine interface. J Clin Invest.

[B58] Naka T, Fujimoto M, Tsutsui H, Yoshimura A (2005). Negative regulation of cytokine and TLR signalings by SOCS and others. Adv Immunol.

[B59] Bode JG, Nimmesgern A, Schmitz J, Schaper F, Schmitt M, Frisch W, Haussinger D, Heinrich PC, Graeve L (1999). LPS and TNFalpha induce SOCS3 mRNA and inhibit IL-6-induced activation of STAT3 in macrophages. FEBS Lett.

[B60] Yadav A, Kalita A, Dhillon S, Banerjee K (2005). JAK/STAT3 pathway is involved in survival of neurons in response to insulin-like growth factor and negatively regulated by suppressor of cytokine signaling-3. J Biol Chem.

[B61] Breitner JC, Welsh KA, Helms MJ, Gaskell PC, Gau BA, Roses AD, Pericak-Vance MA, Saunders AM (1995). Delayed onset of Alzheimer's disease with nonsteroidal anti-inflammatory and histamine H2 blocking drugs. Neurobiol Aging.

[B62] in t' Veld BA, Ruitenberg A, Hofman A, Launer LJ, van Duijn CM, Stijnen T, Breteler MM, Stricker BH (2001). Nonsteroidal antiinflammatory drugs and the risk of Alzheimer's disease. N Engl J Med.

[B63] Stewart WF, Kawas C, Corrada M, Metter EJ (1997). Risk of Alzheimer's disease and duration of NSAID use. Neurology.

[B64] Aisen PS (2002). Evaluation of selective COX-2 inhibitors for the treatment of Alzheimer's disease. J Pain Symptom Manage.

[B65] Aisen PS, Schafer KA, Grundman M, Pfeiffer E, Sano M, Davis KL, Farlow MR, Jin S, Thomas RG, Thal LJ (2003). Effects of rofecoxib or naproxen vs placebo on Alzheimer disease progression: a randomized controlled trial. Jama.

[B66] Reines SA, Block GA, Morris JC, Liu G, Nessly ML, Lines CR, Norman BA, Baranak CC (2004). Rofecoxib: no effect on Alzheimer's disease in a 1-year, randomized, blinded, controlled study. Neurology.

[B67] Thal LJ, Ferris SH, Kirby L, Block GA, Lines CR, Yuen E, Assaid C, Nessly ML, Norman BA, Baranak CC, Reines SA (2005). A randomized, double-blind, study of rofecoxib in patients with mild cognitive impairment. Neuropsychopharmacology.

[B68] Rogers J, Kirby LC, Hempelman SR, Berry DL, McGeer PL, Kaszniak AW, Zalinski J, Cofield M, Mansukhani L, Willson P (1993). Clinical trial of indomethacin in Alzheimer's disease. Neurology.

